# Mechanistic Effects of Amino Acids and Glucose in a Novel Glutaric Aciduria Type 1 Cell Model

**DOI:** 10.1371/journal.pone.0110181

**Published:** 2014-10-15

**Authors:** Xi Fu, Hongjie Gao, Fengyan Tian, Jinzhi Gao, Liping Lou, Yan Liang, Qin Ning, Xiaoping Luo

**Affiliations:** 1 Department of Pediatrics, Tongji Hospital, Tongji Medical College, Huazhong University of Science and Technology, Wuhan, China; 2 Department of Pathology, Tongji Hospital, Tongji Medical College, Huazhong University of Science and Technology, Wuhan, China; 3 Department of Infectious Diseases, Tongji Hospital, Tongji Medical College, Huazhong University of Science and Technology, Wuhan, China; Massachusetts General Hospital, United States of America

## Abstract

Acute neurological crises involving striatal degeneration induced by a deficiency of glutaryl-CoA dehydrogenase (GCDH) and the accumulation of glutaric (GA) and 3-hydroxyglutaric acid (3-OHGA) are considered to be the most striking features of glutaric aciduria type I (GA1). In the present study, we investigated the mechanisms of apoptosis and energy metabolism impairment in our novel GA1 neuronal model. We also explored the effects of appropriate amounts of amino acids (2 mM arginine, 2 mM homoarginine, 0.45 g/L tyrosine and 10 mM leucine) and 2 g/L glucose on these cells. Our results revealed that the novel GA1 neuronal model effectively simulates the hypermetabolic state of GA1. We found that leucine, tyrosine, arginine, homoarginine or glucose treatment of the GA1 model cells reduced the gene expression of caspase-3, caspase-8, caspase-9, bax, fos, and jun and restored the intracellular NADH and ATP levels. Tyrosine, arginine or homoarginine treatment in particular showed anti-apoptotic effects; increased α-ketoglutarate dehydrogenase complex (OGDC), fumarase (FH), and citrate synthase (CS) expression; and relieved the observed impairment in energy metabolism. To the best of our knowledge, this study is the first to investigate the protective mechanisms of amino acids and glucose in GA1 at the cellular level from the point of view of apoptosis and energy metabolism. Our data support the results of previous studies, indicating that supplementation of arginine and homoarginine as a dietary control strategy can have a therapeutic effect on GA1. All of these findings facilitate the understanding of cell apoptosis and energy metabolism impairment in GA1 and reveal new therapeutic perspectives for this disease.

## Introduction

The mitochondrial catabolism of lysine and tryptophan is disrupted by a deficiency of glutaryl-coenzyme A dehydrogenase (EC 1.3.99.7) (GCDH), causing glutaric aciduria type 1 (GA1) (OMIM #231670), which leads to the accumulation of glutaryl-CoA, glutaric acid (GA), 3-hydroxyglutaric acid (3-OHGA) and glutaconic acid (GC) in body fluids and tissues [1]–[4]. Elevated levels of GA and 3-OHGA may cause neuronal damage by blocking the tricarboxylic acid cycle (TCA cycle) [5]. Additionally, glutaryl-CoA can inhibit the activity of the α-ketoglutarate dehydrogenase complex (OGDC), which is the rate-limiting enzyme in the TCA cycle [6]. GA1, first reported by Goodman in the 1970s, is a common human autosomal recessive inherited metabolic disorder [7], [8]. Patients affected with GA1 usually present with an encephalopathic crisis that involves typical striatal injury. GA1 may be triggered by a catabolic state or stress, such as inflammation, vaccination or surgery, and the affected individual may subsequently develop neurological/behavioral abnormalities by the age of two years [9].

Appropriate therapy for GA1 includes dietary restriction and control, carnitine/riboflavin supplementation and emergency treatment [10]. Dietary restriction and control are especially crucial for reducing GA-producing substrates and minimizing the risk of adverse neurological outcomes [11], [12]. Supplementation of arginine or glucose alone in the regimen could significantly increase the survival of GCDH-deficient (Gcdh–/–) mice receiving a high-lysine diet. An even higher survival rate was observed using a diet containing arginine with glucose [13], [14]. Furthermore, the protective effect of combined treatment with homoarginine and glucose is strong, resulting in 100% survival in the GA1 mouse model [15]. Based on these studies, a novel treatment strategy was developed using lysine-free, arginine-fortified amino acid supplements in light of the competitive effect of arginine on brain lysine uptake at the blood-brain barrier [16], [17]. The metabolic crises observed in GA1 patients always cause hypoglycemia, which in turn results in amino acid uptake and altered gluconeogenesis function in the liver [18]. In the immature brain, a reduced glucose level causes elevated consumption of alternate energy substrates, such as ketone bodies, and therefore increases the breakdown of ketogenic amino acids such as lysine and branched-chain amino acids (BCAAs) [15]. The activities of lysine metabolism-related transporters and enzymes decrease with brain maturation; thus, the immature brain is more likely to be affected by amino acid metabolism disorders [19], [20]. Consequently, glucose administration may prevent and reduce striatal injury in human GA1 encephalopathy. In a Gcdh–/– mouse model fed a high-lysine diet, glucose not only corrects hypoglycemia and reduces the demand for alternate substrates but also lowers brain lysine uptake, a rate-limiting step of lysine metabolism [13]. However, more work is required to gain insight into the effect and mechanisms of arginine, homoarginine and glucose on GA1 at the cellular level. Various important biochemical activities in the brain are often related to BCAAs, including leucine [21]. Leucine passes more rapidly through the blood-brain barrier than any other amino acid [22], [23], and it might therefore have priority in entering the brain and protecting it from the impact of other amino acids. Additionally, the breakdown of leucine generates acetyl coenzyme A (acetyl-CoA) to generate energy in the TCA cycle. Nearly half of the α-amino groups of brain glutamate are derived from leucine alone [24]. Glutamate is vital for maintaining brain activities. However, it can also induce neuronal toxicity when present in excessive levels. Tyrosine can enter the TCA cycle via both acetyl-CoA and fumarate [25]. Additional investigations are warranted to determine whether supplementation of leucine, tyrosine, arginine, homoarginine or glucose can compensate for the energy deficiency observed in GA1 or whether it might induce further cytotoxicity.

Since Fire and Mello discovered RNA interference (RNAi) in 1998, RNAi technology has been widely applied to various fields, including the exploration of gene function, gene therapy, and the treatment of neoplastic diseases and genetic diseases because RNAi can be used to specifically suppress the expression of target genes [26]–[28]. Viral vector systems carrying short hairpin RNA (shRNA) fragments can induce effective target gene interruption and thereby achieve gene knockdown. Lentivirus vectors, which are one of the most widely used types of vectors for exploring genetic diseases involving neurodegeneration, can maintain stable and durable target gene suppression in non-dividing cells, including neurons [26], [27], [29]. We recently established novel GA1 model cells in which we used lentivirus-mediated shRNA to knock down the GCDH gene in rat striatal neurons and subsequently cultured them in lysine-rich medium to simulate striatal degeneration similar to that observed in human GA1 encephalopathic crisis [30].

In this study, we validated the underlying mechanism of GA1-induced striatal degeneration via GCDH gene silencing and lysine intervention. We also explored whether appropriate concentrations of leucine, tyrosine, arginine, homoarginine and glucose have neuroprotective effects on GA1 model cells by assessing nuclear morphology, apoptosis, the intracellular levels of ATP and the reduced form of nicotinamide adenine dinucleotide (NADH), and the expression levels of key enzymes involved in the apoptosis pathway and the TCA cycle.

The information gathered from this study provides guidance and new perspectives for future dietary therapy for GA1.

## Materials and Methods

### Primary neuronal cultures and transduction

Primary striatal neuronal cultures were prepared from neonatal rats (Sprague-Dawley) at postnatal day 1 following decapitation using surgical scissors to minimize animal suffering. All of the experiments were performed with the approval of the Animal Experiments Ethics Committee of Tongji Medical College (Permit Number 2011–S248). The guidelines for the Care and Use of Laboratory Animals from the National Institutes of Health were strictly followed. Striatal tissue was isolated and cut into 1-mm^3^ fragments and incubated with 2 mg/mL papain (Sigma-Aldrich, St. Louis, MO, USA) for 20 min at 37°C to continue digestion. Neurons were then plated at a density of 5×10^5^ cells/well in 6-well plates (for the identification of neurons and protein isolation) or 5×10^4^ cells/well in 96-well plates (for CCK-8 detection) coated with 30 µg/mL poly-D-lysine and 2 g/mL laminin (Sigma-Aldrich) in DMEM/F12 (Hyclone, Logan, UT, USA) containing 10% horse serum (Hyclone) and 10% fetal bovine serum (Gibco, Grand Island, NY, USA). After 6 h, the cells were grown in maintenance medium consisting of neurobasal A medium (Gibco) supplemented with 2% B27 (Gibco) and 0.5 mM glutamine (Sigma-Aldrich) at 37°C under 5% CO_2_. The medium was renewed every other day.

The cultures were washed using 1 mM PBS and fixed with 4% paraformaldehyde (Sigma-Aldrich). After permeabilization with 0.3% TritonX-100 (Sigma-Aldrich), the fixed cultures were incubated with a polyclonal rabbit anti-MAP2 antibody (1∶1,000, Abcam, Cambridge, UK) at 4°C overnight. After a 2-hour incubation using a Cy3-conjugated anti-rabbit antibody (1∶250, Sigma-Aldrich), the nuclei were then stained using Hoechst 33342 (5 µg/mL, Sigma-Aldrich) in the dark for 6 min. To assess the purity of the neurons, the stained cells were washed, subjected to fluorescence microscopy (Olympus BX51 AX-70, Tokyo, Japan), and analyzed using Image-Pro plus 6.0 (Media Cybernetics, Bethesda, MD, USA). A lentivirus-mediated short hairpin RNA (shRNA) targeting the rat GCDH gene (lentivirus-shRNA, Genechem, Shanghai, China) was successfully constructed in a previous study [30]. The primary striatal neurons were maintained in maintenance medium for 5 days before transduction. Transduction was conducted as described by Gao et al. [30] The transduction efficiency was assessed using fluorescence microscopy at 72 h after the transduction of the primary cells with a negative control (NC) lentivirus (Genechem) containing an empty vector that expresses enhanced green fluorescent protein (eGFP) at a multiplicity of infection (MOI)  = 10 (Figure S1). To generate GA1 model cells, the primary striatal neurons were transduced using lentivirus-shRNA (MOI  = 10). After growing the cells for 72 h, they were incubated in maintenance medium with 5 mM lysine for an additional 24 h.

### Study design

Glucose, lysine, leucine, arginine, homoarginine and tyrosine powders were all obtained from Sigma-Aldrich, and solutions with the appropriate concentrations were prepared using maintenance medium for dilution. The GCDH-silenced neurons were incubated in maintenance medium containing 5 mM lysine either alone or in combination with one of the following supplements at the indicated concentrations, including leucine (1, 5, 10, 20, or 50 mM), 0.45 g/L tyrosine, arginine (0.5, 1, 2, 5 or 10 mM), homoarginine (0.5, 1, 2, 5 or 10 mM), or glucose (0.5, 1, 2, 5 or 10 g/L) for 24 h to assess toxicity and to determine the appropriate concentrations of the supplements for further investigations. The optimal concentration of each supplement, namely 10 mmol/L leucine, 0.45 g/L tyrosine, 2 mM arginine, 2 mM homoarginine, or 2 g/L glucose was determined using CCK-8 (selecting for relatively high cell viability) and Hochest staining assay (selecting for relatively less abnormal nuclei).

The NC lentivirus and lentivirus-shRNA infected primary cells grown in maintenance medium with and without 5 mM lysine for 24 h and the lentivirus-shRNA infected primary cells grown in maintenance medium containing 5 mM lysine and one of the supplements with the corresponding optimal concentration for 24 h were harvested. These cells were then subjected to annexin V-PE/7-AAD staining, NADH and ATP assays, real-time RT-PCR and western blot analysis in order to investigate the cell apoptosis and energy impairment of GA1 and explore the potential therapeutic effects of leucine, tyrosine, arginine, homoarginine and glucose on GA1 model cells.

### CCK-8 and Hoechst staining assay

The CCK-8 assay was performed using cell counting kit 8 (Dojindo Molecular Technologies, Gaithersburg, MD, USA) according to the manufacturer's instructions. Optical density (OD) was measured at 450 nm using a spectrophotometer. Cell viability was calculated according to the following equation: Cell viability (%)  =  (OD of GA1 model cells with different treatments – OD of blank control)/(OD of cells with NC lentivirus infection and 5 mM lysine incubation – OD of blank control) ×100.

The Hoechst staining assay was performed by incubating the cells in Hoechst 33342 (1∶10,000) (Sigma-Aldrich) at 37°C for 15 min and then fixing the cells for 15 min using 4% paraformaldehyde. Nuclear morphology was evaluated using fluorescent microscopy (Ex = 350 nm; Em = 460 nm).

### Annexin V-PE/7-AAD staining

The cells were harvested via trypsinization and washed in cold PBS (mixed with 1.5% bovine serum albumin) twice. Subsequently, the resuspended cells (5×10^5^ cells/mL in 1× binding buffer) were stained using Annexin V-PE and 7-AAD, in accordance with the manufacturer's instructions for the Annexin V-PE/7-AAD apoptosis kit (MultiSciences Biotech Co., Ltd., Hangzhou, China). After staining, the number of apoptotic cells in each group was analyzed through flow cytometry (BD Biosciences, San Jose, CA, USA).

### NADH and ATP assays

A total of 2×10^5^ or 1×10^6^ cells were harvested for each NADH or ATP assay, respectively. Both assays were performed according to the manufacturer's instructions for the NAD^+^/NADH quantification colorimetric kit (BioVision, Mountain View, CA) and the ATP colorimetric/fluorometric assay kit (BioVision). Using a spectrophotometer, the intracellular levels of NADH and ATP in the samples were determined based on pre-defined standard curves.

### Real-time RT-PCR

Total RNA was extracted from the striatal neurons plated in 6-well plates using TRIzol (Invitrogen, Life Technologies, Carlsbad, CA, USA). The procedures for cDNA synthesis and real-time PCR amplification were performed according to the descriptions of Gao et al. [30]. The primer sequences designed using Primer 5.0 (synthesized by Invitrogen, China) are shown in Table S1 in File S1.

### Western blot analysis

Protein was extracted from the cells seeded in 6-well plates using cell lysis buffer (Beyotime, China) and quantified using a BCA protein assay kit (Beyotime). After denaturation, the protein samples (30 µg/lane) were prepared for subsequent electrophoresis, transfer, blocking and incubation of antibodies, as described by Gao et al. [30]. Antibodies against caspase-8 (1∶1000, Cell Signaling Technology, Danvers, MA, USA), caspase-9 (1∶1,000, Cell Signaling Technology), fumarase (FH) (1∶1000, Cell Signaling Technology), jun (1∶1500, Abcam), GADPH (1∶1500, Cell Signaling Technology), fos (1∶500, Abcam), the α-ketoglutarate dehydrogenase complex (OGDC) (1∶500, Santa Cruz Biotechnology, Paso Robles, CA, USA), citrate synthase (CS) (1∶500, Santa Cruz Biotechnology), caspase-3 (1∶150, Abcam), bcl2 (1∶150, Santa Cruz Biotechnology), and bax (1∶150, Santa Cruz Biotechnology) were diluted in 5% skim milk powder with 0.2% PBS-Tween 20.

### Statistical analysis

The data were derived from three independent experiments (mean ± standard deviation). Statistical analysis was performed by comparing the GA1 model cells with other conditions using two-way analysis of variance (ANOVA). Moreover, Bonferroni's multiple comparison test (comparison of multiple groups) was conducted to compare cells infected with the NC lentivirus or lentivirus-shRNA and then incubated with or without 5 mM lysine using GraphPad Prism (v.5). *P*<0.05 was considered to be statistically significant.

## Results

### Viability and apoptosis of the cell cultures

In cultured striatal neurons, cell viability, as determined using trypan blue exclusion tests, was greater than 95%. Additionally, more than 90% of living cells were found to be MAP2-positive based on immunofluorescence (Figure S2). The toxicity test for assessing the effects of different supplements on GA1 model cells was performed using the CCK-8 assay. The most appropriate concentrations of each cell culture supplement in the tested ranges were 10 mM leucine, 0.45 g/L tyrosine, 2 mM arginine, 2 mM homoarginine and 2 g/L glucose, in which the model cell cultures exhibited the highest cell viabilities of 56.9%, 57.1%, 67%, 60.1% and 56.5%, respectively. The cells growing in the most appropriate concentrations of all of these supplements exhibited higher cell viability than the GA1 model cells (Table S2 in File S1).

**Figure 1:**
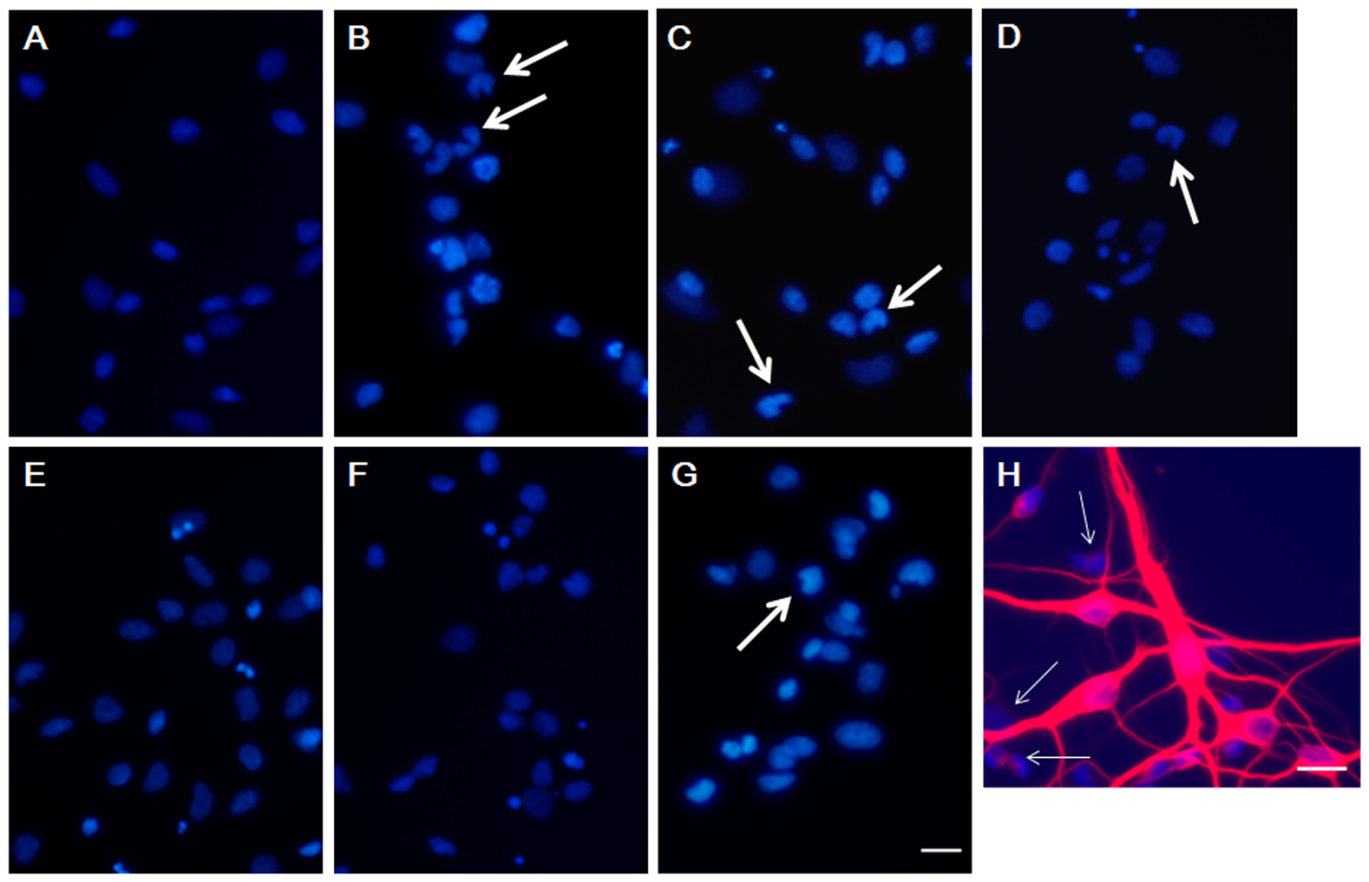
Hoechst 33342 staining of apoptotic striatal neurons. The effects of leucine, tyrosine, arginine, homoarginine and glucose treatment on the nuclear morphology in GA1 model cells. The abnormal morphology of the crescent-shaped nuclei is indicated with wide arrows; the disappeared synapses in GA1 neuronal model cells with crescent-shaped nuclei are indicated with thin arrows. (A) NC+5 mM lysine, (B) LV-shRNA+5 mM lysine (GA1 model cells), (C) GA1 model cells +10 mM leucine, (D) GA1 model cells +0.45 g/L tyrosine, (E) GA1 model cells +2 mM arginine, (F) GA1 model cells +2 mM homoarginine, (G) GA1 model cells +2 g/L Glucose, (H) a merged image showing GA1 model cells stained with Hoechst 33342 (nuclei) and MAP-2 (neuronal bodies and dendrites). Scale bars  = 20 µm.

Following nuclear staining using Hoechst 33342, neuronal nuclei exhibiting an apoptosis-related morphology, such as shrunken and irregularly shaped nuclei (crescent-shaped nuclei indicated with wide arrows), were frequently detected in the GA1 model cells, whereas healthy (oval-shaped) nuclei were observed in the NC cells in medium containing 5 mM lysine (Figure 1 A&B). The nuclei of GA1 model cells incubated in 2 mM arginine and 2 mM homoarginine resembled those detected in NC cells in medium containing 5 mM lysine (control cultures), which suggests that the use of the tested supplements, especially arginine and homoarginine, appeared to reduce the number of nuclei with an abnormal morphology among GA1 model cells (Figure 1 B, E&F). A merged image (Figure 1 H) of Hoechst 33342 staining and MAP2 labeling revealed GA1 neuronal model cells whose synapses had disappeared with crescent-shaped nuclei (indicated with thin arrows). The cells showing an abnormal nuclear morphology were nearly incapable of developing dendrites, which implied that they may have been “injured” cells.

**Figure 2:**
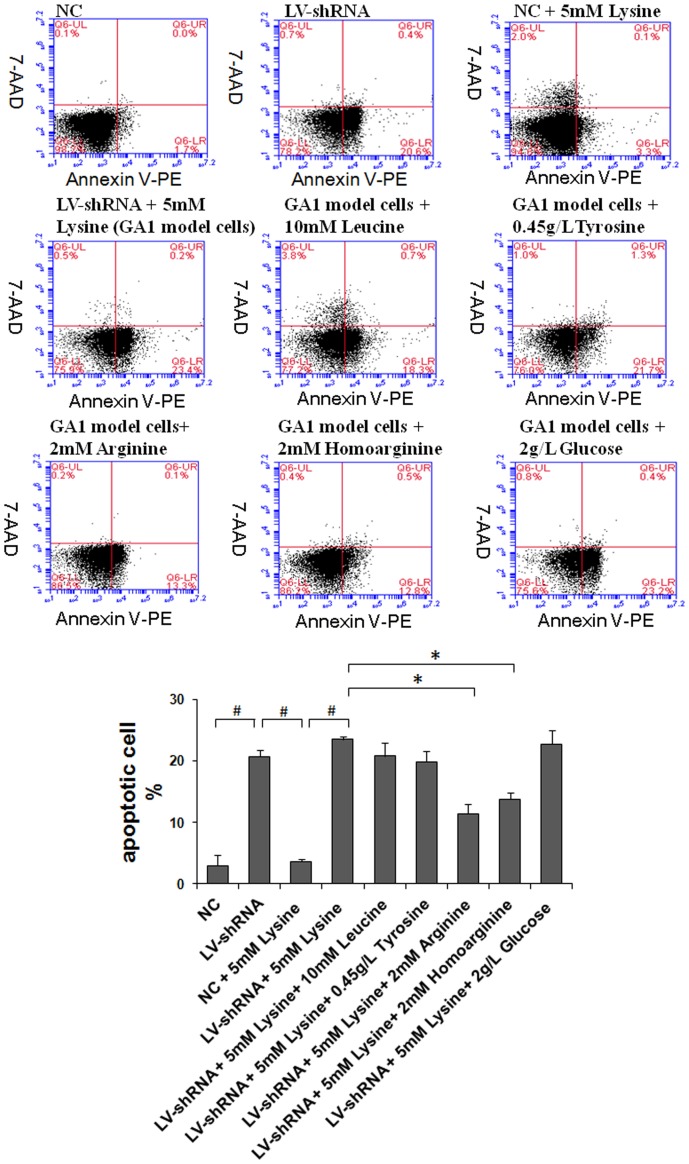
Detection of apoptosis using flow cytometry. Cell apoptosis was evaluated using Annexin V-PE/7-AAD staining and flow cytometry. The lentivirus-shRNA (LV-shRNA) group showed significantly induced apoptosis, whereas treatment with lysine slightly increased the rate of apoptosis. However, this increase was not statistically significant. The arginine, homoarginine treatment significantly reduced the apoptosis of GA1 model cells, whereas the leucine and tyrosine treatment led to a slightly decrease in apoptosis that was not statistically significant. The data are shown as the mean ± standard deviation. An asterisk (*) indicates *P*<0.05 using two-way ANOVA. A pound sign (^#^) indicates *P*<0.05 using Bonferroni's multiple comparison test. *n* = 3.

The number of apoptotic cells was determined using Annexin V-PE/7-AAD staining and flow cytometry (Figure 2). The frequency of apoptosis was high in lentivirus-shRNA cells without lysine treatment and was further increased in the GA1 model cells. Therefore, down-regulation of the GCDH gene using lentivirus-shRNA in striatal neurons induced apoptosis, and treating the cells with lysine exacerbated this effect. The supplementation of 2 g/L glucose did not change the apoptotic cell fraction, whereas supplementation of 2 mM arginine or homoarginine significantly reduced apoptosis in the GA1 model neurons. Furthermore, GA1 model cells exposed to medium containing 10 mM leucine or 0.45 g/L tyrosine also showed a slight reduction in apoptosis. In view of these findings, amino acids including arginine, homoarginine, leucine and tyrosine may have anti-apoptotic effects on GA1 model cells.

### Expression of apoptosis-related genes and proteins

**Figure 3:**
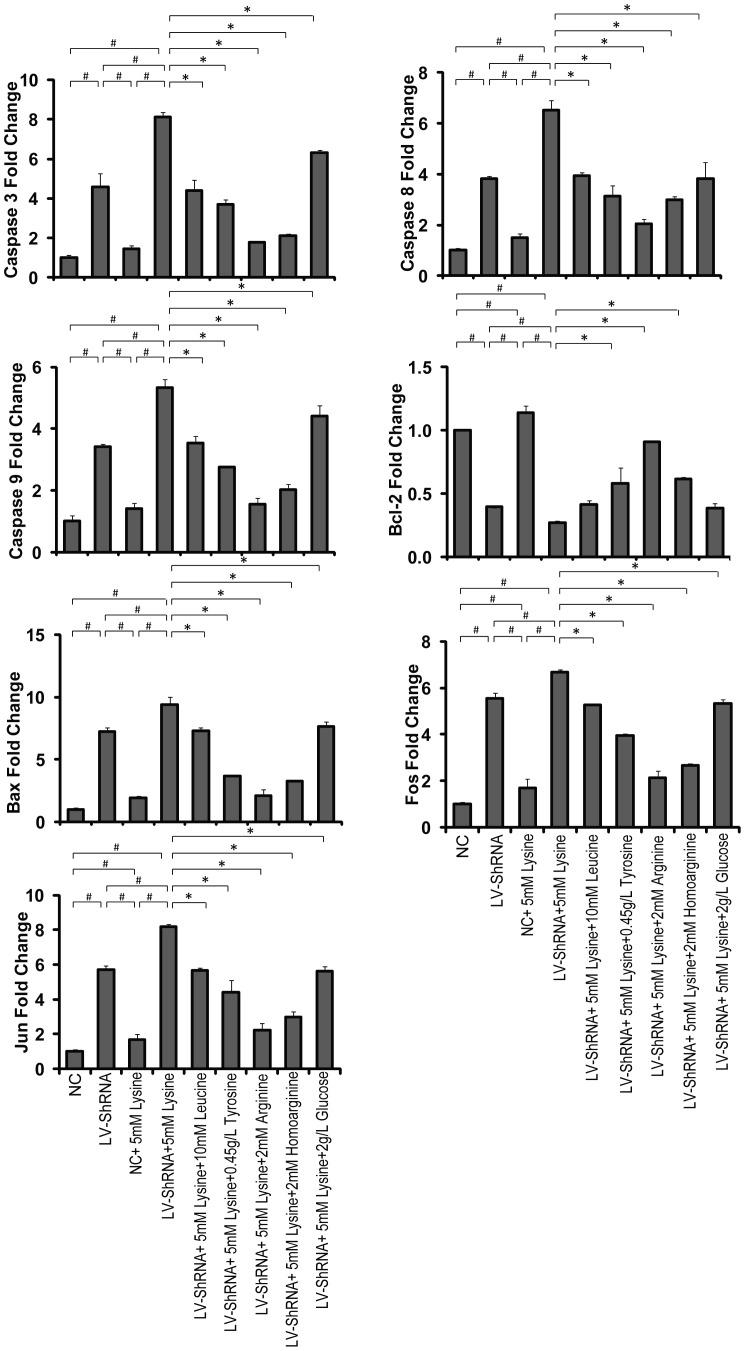
Expression of apoptosis-related genes. The transcript levels of caspases 3, 8, and 9; bax; fos and jun were significantly up-regulated in lentivirus-shRNA (LV-shRNA) group, whereas the bcl-2 transcript level was down-regulated. When combining with the lysine treatment, these regulations were intensified. The leucine, tyrosine, arginine, homoarginine and glucose treated groups showed significantly down-regulated the transcript levels of caspases 3, 8, and 9; bax; fos and jun. The bcl-2 gene expression was significantly increased only in the tyrosine, arginine, and homoarginine treated groups. The data are shown as the mean ± standard deviation. An asterisk (*) indicates *P*<0.05 using two-way ANOVA. A pound sign (^#^) indicates *P*<0.05 using Bonferroni's multiple comparison test. *n* = 3.

**Figure 4:**
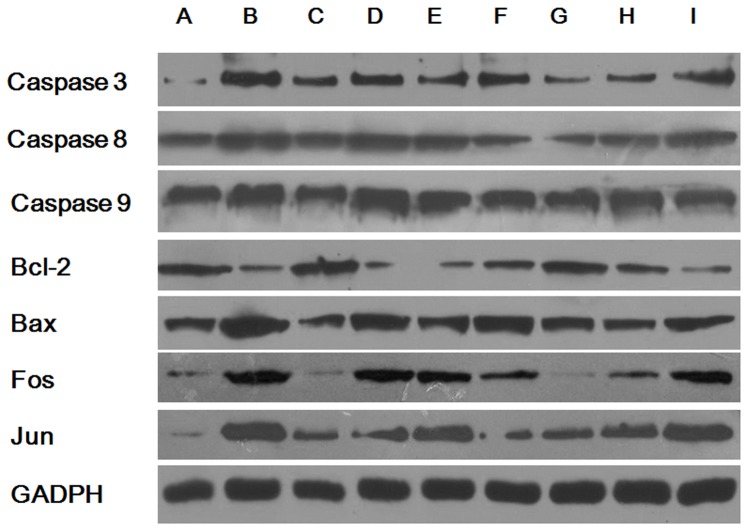
Expression of apoptosis-related proteins. (A) NC, (B) LV-shRNA+5 mM lysine (GA1 model cells), (C) NC+5 mM lysine, (D) LV-shRNA, (E) GA1 model cells +10 mM leucine, (F) GA1 model cells +0.45 g/L tyrosine, (G) GA1 model cells+2 mM arginine, (H) GA1 model cells +2 mM homoarginine, (I) GA1 model cells +2 g/L glucose. Similar trends were observed in the expression of apoptosis-related proteins compared to the expression of the corresponding apoptosis-related genes, with the exceptions of caspase 8 and caspase 9, bcl-2 (between group B and D), fos (between group A and C), and jun (between group B and I).

The abnormal metabolites in GA1 can trigger partially caspase-dependent neuronal apoptosis [30], therefore to explore more apoptosis-related genes and proteins become necessary in order to gain a more complete view of GA1 related apoptosis. The expression levels of apoptosis-related genes and proteins were analyzed using real-time PCR and western blot analyses, respectively (Figure 3 & 4). The gene levels of caspase-3, caspase-8, caspase-9, bax, fos and jun were significantly up-regulated, whereas bcl-2 was down-regulated in cells transduced with lentivirus-shRNA without lysine treatment. Enhanced effects on these genes and proteins showing similar trends were also observed in the GA1 model cells. No change in the expression of apoptosis-related genes was shown in cells transduced with the NC lentivirus, regardless of lysine treatment. Treatment of the GA1 model cells using leucine and glucose, tyrosine, arginine and homoarginine significantly down-regulated the gene expression levels of caspase-3, caspase-8, caspase-9, bax, fos, and jun. Additionally, supplementation of tyrosine, arginine and homoarginine in the cultures of GA1 model cells up-regulated bcl-2 gene expression. Similar trends were found in apoptosis-related protein expression to those observed for gene expression, with only a few exceptions. Caspase-8 and caspase-9 gene expressions were different from that recorded in previous studies [30]. The finding that the levels of mRNA transcription do not correlate with the levels of protein expression may be attributed to the underlying post-transcriptional and post-translational regulatory mechanisms. Additionally, the protein expression (Figure 4) of bcl-2 (between group B and D), fos (between group A and C), and jun (between group B and I) do not correlate with the levels of their gene expression (Figure 3). This may be attributed to experimental error of western blot.

### Intracellular levels of NADH and ATP

**Figure 5:**
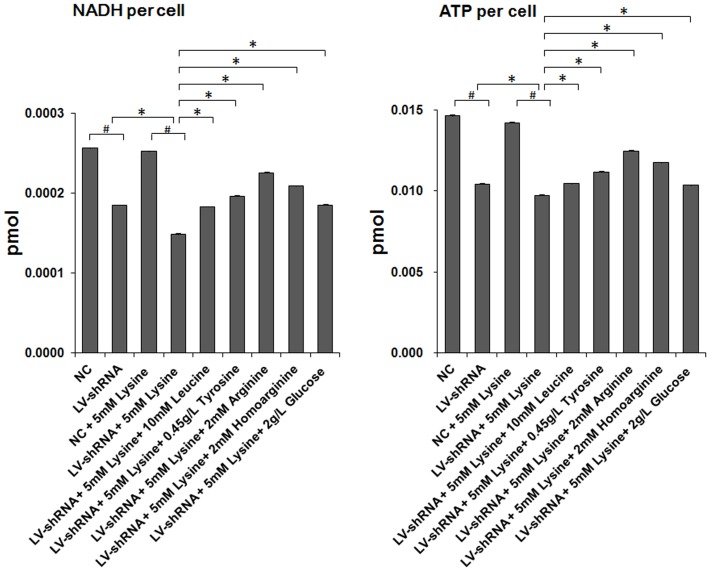
Intracellular levels of NADH and ATP. The intracellular levels of NADH and ATP were evaluated through chemical colorimetric detection. The intracellular NADH and ATP levels were significantly reduced in lentivirus-shRNA (LV-shRNA) group. These effects were intensified by the lysine treatment. The leucine, tyrosine, arginine, homoarginine and glucose treated groups showed significantly increase in the intracellular NADH and ATP levels. The data are shown as the mean ± standard deviation. An asterisk (*) indicates *P*<0.05 using two-way ANOVA. A pound sign (^#^) indicates *P*<0.05 using Bonferroni's multiple comparison test. *n* = 3.

Impairment of energy metabolism caused by GA1 is one of the most important causes of striatal injury and neuronal dysfunction. In the present study, we evaluated the intracellular levels of NADH and ATP in cell cultures subjected to different treatments using chemical colorimetric detection, as shown in Figure 5. Both NADH and ATP levels were significantly reduced in the primary neurons infected with lentivirus-shRNA alone. These reductions were enhanced in the GA1 model cells when lentivirus-shRNA infection was combined with lysine treatment. These findings revealed that GCDH gene silencing alone can cause impairment of energy generation, and lysine treatment of these cells can aggravate this effect. Furthermore, the intracellular levels of NADH and ATP observed in GA1 model cells were significantly increased through leucine, tyrosine, arginine, homoarginine or glucose treatment, although they could not be fully restored to a normal state. Arginine, homoarginine or tyrosine treatment, in particular, improved the energy generation capability of the GA1 model cells over that observed in the primary neurons infected by lentivirus-shRNA alone.

### Expression of TCA cycle-related enzymes

**Figure 6:**
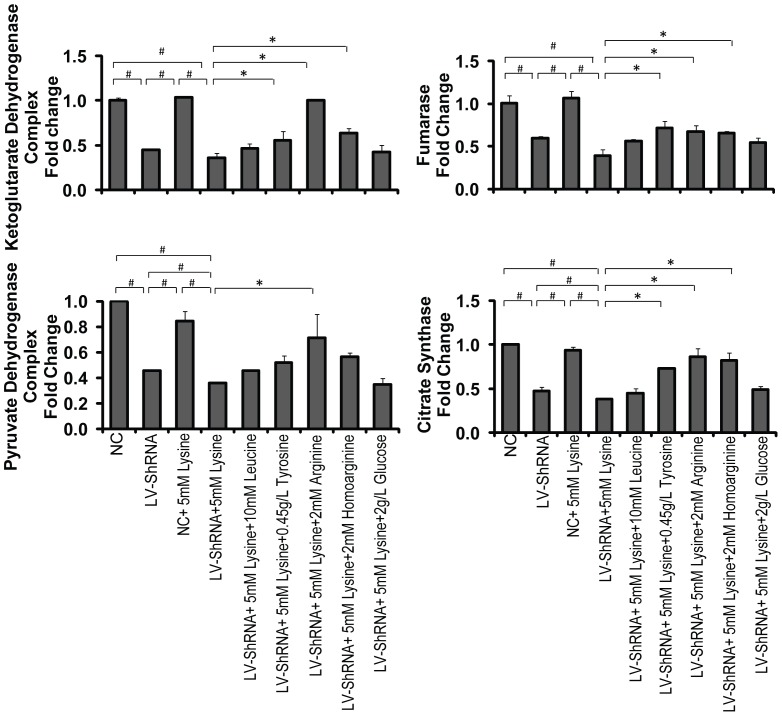
Gene expression of TCA cycle-related enzymes. The transcript levels of OGDC, FH, PDC and CS were significantly down-regulated in lentivirus-shRNA (LV-shRNA) group. When combining with lysine treatment, the down-regulation of PDC and CS was intensified. The treatment of tyrosine, arginine and homoarginine significantly up-regulated the expression of OGDC, FH and CS genes. PDC was up-regulated only in the arginine treated group. The data are shown as the mean ± standard deviation. An asterisk (*) indicates *P*<0.05 using two-way ANOVA. A pound sign (^#^) indicates *P*<0.05 using Bonferroni's multiple comparison test. *n* = 3.

**Figure 7:**
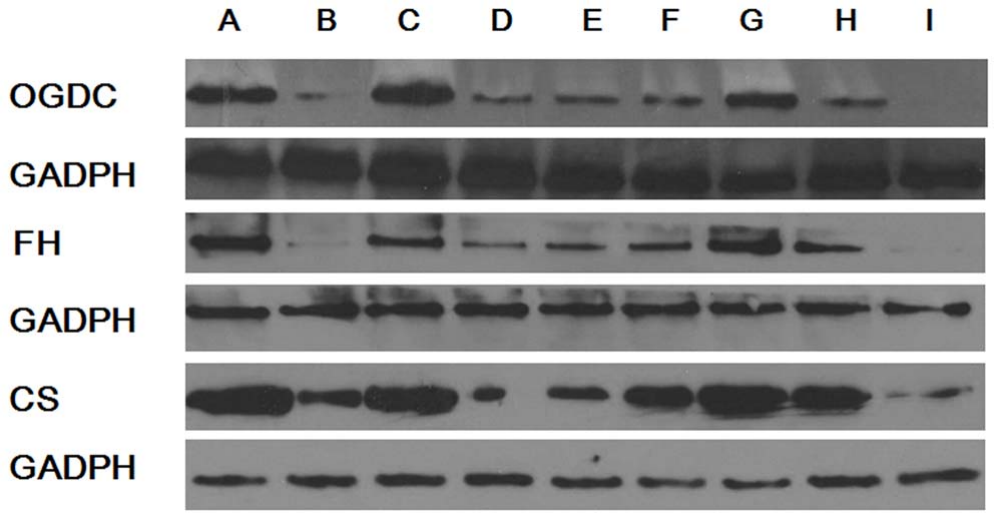
Expression of TCA cycle-related proteins. (A) NC, (B) LV-shRNA+5 mM lysine (GA1 model cells), (C) NC+5 mM lysine, (D) LV-shRNA, (E) GA1 model cells +10 mM leucine, (F) GA1 model cells +0.45 g/L tyrosine, (G) GA1 model cells+2 mM arginine, (H) GA1 model cells +2 mM homoarginine, (I) GA1 model cells +2 g/L glucose. Similar trends were observed in the expression of TCA cycle-related proteins and the expression of the corresponding genes encoding TCA cycle-related enzymes, with the exception of CS (between group B and D).

To fully understand energy metabolism and the effect of our selected supplements in the cultures of GA1 model cells, we investigated several rate-limiting enzymes in the TCA cycle, including OGDC, FH, PDC and CS (Figures 6 & 7). The gene expression of OGDC, FH, PDC and CS was significantly down-regulated in the primary neurons via lentivirus-shRNA transduction. Combining lentivirus-shRNA transduction with lysine treatment in the primary neurons further reduced the gene expression of PDC and CS, which demonstrated that GCDH gene silencing can cause TCA cycle disorders. This effect was enhanced when combined with lysine treatment. The tyrosine, arginine and homoarginine treatment of GA1 model cells up-regulated the gene expression of OGDC, FH and CS, with significant differences being detected. However, up-regulation of PDC gene expression in GA1 model cells was only observed when the cells were supplemented with arginine. Notably, arginine treatment was able to restore the gene expression of OGDC, CS and PDC, and homoarginine treatment was also able to restore CS gene expression to levels that were not significantly different from the normal state. The expression of TCA cycle-related enzymes assessed using western blot analysis was, to a considerable extent, consistent with the results of the gene expression analysis using real-time PCR. Although a few exceptions were observed, such as the results for CS (between group B and D), we believe that these discrepancies may primarily be attributed to experimental error because the western blot analysis was conducted without replicates. The results revealed that arginine and homoarginine can improve energy metabolism and partially compensate for the down-regulation of these enzymes in GA1 model cells.

## Discussion

**Figure 8:**
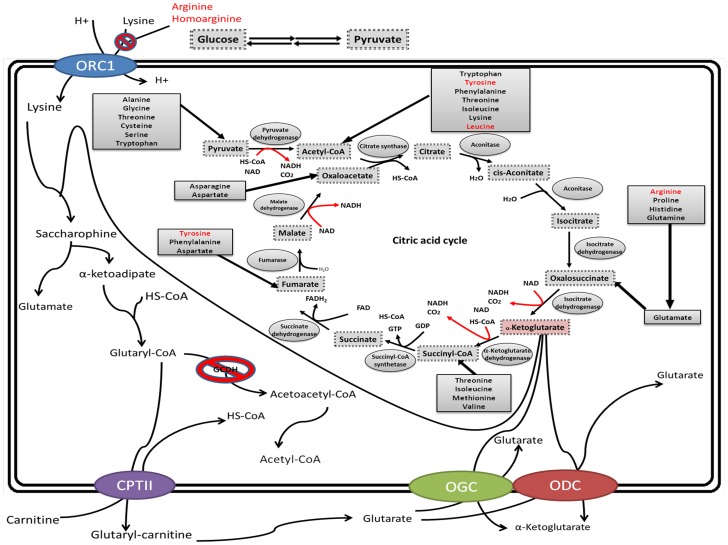
Proposed mechanism underlying the effects of leucine, tyrosine, arginine and homoarginine on the regulation of energy metabolism in GA1 cells. Lysine is first transported into the mitochondria via the ornithine carrier (ORC1) and subsequently degraded. Reacting with α-ketoglutarate (α-KG), lysine is converted to saccharopine, subsequently generating glutaryl-CoA, which can be catalyzed by GCDH to form acetyl-CoA. A defect in GCDH leads to glutaryl-CoA and GA accumulation. The counter-exchange of accumulated GA and α-KG through the oxodicarboxylate carrier (ODC) and oxoglutarate carrier (OGC) results in extreme depletion of α-KG. The reaction converting α-KG to succinyl-CoA in the TCA cycle is hindered; thus, the regeneration of oxaloacetate (OAA) is limited. The insufficient supply of OAA restricts acetyl-CoA from entering the TCA cycle. Acetyl-CoA is highly accumulated, and the TCA cycle is interrupted [15]. The oxoglutarate dehydrogenase complex (OGDC) is located in the mitochondrial matrix and mediates the transformation of α-KG to succinyl-CoA and carbon dioxide in the TCA cycle [32], [33]. Fumarase (FH), which is a mitochondrial enzyme, catalyzes fumarate to malate in the TCA cycle [34], [35]. The pyruvate dehydrogenase complex (PDC) is able to channel glycolysis into the TCA cycle as a result of converting pyruvate to acetyl-CoA [36]. Acetyl-CoA can subsequently be converted to citrate in the presence of CS [37]. Leucine, tyrosine and glucose can be converted to acetyl-CoA and enter the TCA cycle. Tyrosine can also generate fumarate. Arginine and homoarginine may inhibit mitochondrial transport of lysine at the mitochondrial carrier (ORC1).

Zinnanti et al. [15], [31] previously suggested that the process of lysine degradation and GA generation occurs primarily in the mitochondria of neurons through the saccharopine pathway (Figure 8). Lysine is first transported into the mitochondria via the ornithine carrier (ORC1) and is subsequently degraded. Reacting with α-ketoglutarate (α-KG) in the mitochondria, lysine is converted to saccharopine, which is further dehydrogenated and oxidized to α-ketoadipate. In the presence of free coenzyme A (CoA), α-ketoadipate is converted to glutaryl-CoA, which can then be catalyzed by GCDH to form acetyl-CoA. When lysine degradation is interrupted by GCDH defects, glutaryl-CoA and, subsequently, GA accumulate at high levels in cells. The counter-exchange of accumulated GA and α-KG through the oxodicarboxylate carriers (ODCs) and oxoglutarate carriers (OGCs) located in the mitochondrial membrane results in extreme depletion of α-KG. Consequently, the reaction converting α-KG to succinyl-CoA in the TCA cycle is hindered, and thus, the regeneration of oxaloacetate (OAA) is limited. An insufficient supply of OAA limits the entry of acetyl-CoA into the TCA cycle, and acetyl-CoA therefore exhibits high accumulation. Consequently, the interrupted TCA cycle will eventually be unable to generate adequate NADH, limiting ATP synthesis [13], [15]. Apart from the saccharopine pathway, Sauer et al. [38] proposed that the peroxisomal pipecolate pathway is the major lysine degradation route in the brain. However, the mechanism that describes how the two pathways work together in the brain has not been established.

According to the literature, GA1-triggered apoptosis in neurons is partially caspase dependent [30]. In the present study, we investigated numerous key molecules involved in several apoptotic pathways. Bcl-2, bax and caspase-9 act as effector molecules in the mitochondrial apoptotic pathway, whereas caspase-8 is crucial for triggering apoptosis via the death receptor-mediated pathway. All of these genes were up-regulated in GA1 model cells. Caspase-3, which integrates signals from its upstream apoptotic pathways and directly controls cell apoptosis, was up-regulated in GA1 model cells [39]. Additionally, c-fos and c-jun are crucial for activating mitogen-activated protein kinases/activator protein 1 (MAPK/AP-1) signaling pathway and, in turn, regulating cell proliferation and apoptosis [40]–[42]. These findings suggest that GA1 induces cell apoptosis via the caspase-8-mediated death receptor pathway as well as through bcl-2-, bax- and caspase-9-mediated mitochondrial pathways. Furthermore, the apoptosis observed in GA1 model cells was likely dependent on the MAPK/AP-1 pathway as well. A previous study demonstrated that apoptosis significantly increases in GCDH-deficient neurons when exposed to excess lysine [30]. However, we did not observe a significant increase of cell apoptosis in this study, although changes in caspase-related genes were observed. The possible reason for this discrepancy may be attributed to the bath-to-batch variation of the primary neurons leading to different response times to lysine in the two studies.

Numerous enzymes involved in energy metabolism were previously demonstrated to exhibit reduced expression and activity in GA1. In rat cortical cultures and midbrain supernatants, the cellular level of creatine kinase, which is vital to ATP regeneration, was shown to be reduced as a result of the accumulation of GA and 3-OHGA [43]–[46]. Additionally, enhanced GA1-related metabolites result in inhibition of the oxoglutarate dehydrogenase complex (OGDC) and disruption of the TCA cycle [5], [6], [47]. In this study, we detected the expression of the pyruvate dehydrogenase complex (PDC), fumarase (FH) and citrate synthase (CS), which are involved in the TCA cycle, and intracellular ATP and NADH levels in well-established lentivirus-mediated GA1 model cells to explore the energy impairment mechanism involved in GA1. We found that the ATP and NADH levels per neuron and the expression of TCA cycle-related enzymes, including OGDC, PDC, FH, and CS, were reduced in GA1 model cells. Therefore, we suggest that GA1 can also limit the expression of the rate-limiting enzymes in the TCA cycle and interrupt the generation of energy.

Amino acid metabolism in the brain could be regulated by various degrees of competitive inhibition at the level of the blood-brain barrier [48]. Leucine has many important biological functions in the brain, including playing roles in the supply of energy and neurotransmitter synthesis [24]. Tyrosine metabolism in the brain generates substrates of the TCA cycle for energy gain. When arginine and homoarginine are used for the treatment of GA1 animal models and patients, it has been proposed that they target both the common cerebrovascular cationic transporter (y+ system) of the blood-brain barrier and ORC1 in the mitochondrial membrane [15]–[17]. Hypoglycemia, which can cause an increase in lysine catabolism during GA1 metabolic crisis, leads to GA accumulation and TCA cycle interruption, resulting in depletion of glutamate, GABA, and ATP followed by neurological damage [18], [49], [50]. Providing a high-glucose diet in a GA1 mouse model can significantly improve animal survival [13]. However, insufficient knowledge has been obtained regarding the action of amino acids and glucose on GA1 at the cellular level.

According to our results, all of the tested supplements, particularly arginine and homoarginine, have been shown to significantly down-regulate the expression of apoptosis-related genes and inhibit the apoptosis of GA1 model cells. We therefore argue that the neuroprotective effect of these supplements on GA1 model cells can be attributed to the inhibition of apoptotic pathways.

Supplementation of tyrosine, arginine, and homoarginine restored the expression of TCA cycle-related enzymes and recovered the observed energy impairment by increasing intercellular ATP and NADH levels. For arginine and homoarginine, these effects most likely occur because they can inhibit the uptake of lysine into the mitochondria by competing for the available ORC1 on the mitochondrial membrane (Figure 8) and likely for other unknown transporters and carriers that are involved in lysine degradation. Furthermore, it is likely that the concentration of α-KG was partly restored in the GA1 model cells subjected to arginine and homoarginine treatment. The reason for this speculation is that the expression of the enzyme that catalyzes a metabolic reaction is often regulated by the intracellular concentration of the corresponding substrate [51]; in our case, the gene expression of OGDC was up-regulated. Notably, although lysine catabolism has been proposed to occur via both the saccharopine pathway and the peroxisomal pipecolate pathway in the brain, the cellular therapeutic mechanism of arginine and homoarginine on GA1 suggested in this study was based on the saccharopine pathway. Whether there is a similar mechanism involved in the peroxisomal pipecolate pathway has not been established, and additional exploration is warranted.

Treatment of GA1 model cells with 2 g/L glucose had a weak effect on reducing apoptosis and recovering the injury to energy metabolism. This observation indicates that glucose can reduce the uptake and catabolism of lysine in the brain *in vivo*, but not *in vitro*, which implies the existence of a neuroprotective effect that corrects hypoglycemia and reduces the demand for alternate substrates [15], [18], which may primarily act at the level of the blood-brain barrier, rather than on individual cells.

This study also demonstrated a weak protective effect of leucine on GA1 model cells. We suggest that the addition of excess leucine to the culture medium of GA1 model cells may cause slight competitive inhibition of lysine uptake because, in some cases, the breakdown of lysine may partly be replaced by other ketogenic amino acids, including leucine, during GA1 metabolic crisis.

Tyrosine, which can be converted to both acetyl-CoA and fumarate and enter into the TCA cycle, displayed a more pronounced therapeutic effect on GA1 model cells. In this case, it is likely that the lack of OAA and the accumulation of acetyl-CoA as the result of α-KG depletion in GA1 model cells can be recovered. Therefore, the set of reactions in the TCA cycle from fumarate via OAA to α-KG, which can produce 2/3 total NADH per TCA cycle, can be partially recovered (Figure 8). Additionally, due to the low solubility of tyrosine, the ability to increase its concentration, which would most likely enhance the neuroprotective effect on GA1 model cells, is limited. Further work on improving tyrosine solubility may increase the potential of tyrosine treatment in GA1 model cells.

## Conclusion and Perspectives

In conclusion, our current findings demonstrated that suppressing the GCDH gene in combination with lysine treatment of striatal neurons may result in TCA cycle disorders and the activation of apoptosis, indicating that our novel GA1 model cells can effectively simulate GA1 metabolic crisis. Treating GA1 model cells with several amino acids, especially tyrosine, arginine and homoarginine, resulted in a neuroprotective effect. The proposed mechanism was based on the effects of both energy impairment recovery and anti-apoptosis. The former effect is a result of the inhibition of mitochondrial transport of lysine by competing against available mitochondrial carriers and supplying the substrates and regulating the expression of enzymes involved in the TCA cycle. The latter effect is demonstrated by the fact that the expression of the genes involved in the mitochondrial and death receptor-mediated apoptotic pathways and MAPK/AP-1 signaling pathway in GA1 model cells was partially restored by treatment with glucose, leucine, tyrosine, arginine and homoarginine, all of which ultimately reduced the number of apoptotic cells. In light of the proposed mechanism, the use of other amino acids or amino acid analogues to generate relevant substrates downstream of α-KG in the TCA cycle, such as supplying succinyl-CoA by isoleucine or OAA by aspartate, may help to correct the impaired functions of the TCA cycle in GA1 cells to a certain degree.

In conclusion, the present study can be used as a reference and provides new perspectives regarding dietary management and therapeutic explorations for patients with GA1. Additionally, the implications derived from and this study may help to develop novel candidate medications and treatment strategies for patients with GA1 in the future.

## Supporting Information

Figure S1 **Striatal neurons infected with lentivirus.** The striatal neurons were infected with the NC lentivirus at an MOI of 10; nearly all of the cells were infected and exhibited normal morphology. The observed fluorescence verified optimal infection conditions (magnification, 200×). (A) Bright-field microscopy of striatal neurons; (B) fluorescence microscopy of striatal neurons.
(TIF)

Figure S2 **Assessment of neuronal purity.** Immunofluorescence staining demonstrated the proportion of neurons in living cells to be greater than 90% (magnification, 200×). (A) Neuronal bodies and dendrites were labeled using Texas Red (red); (B) the cell nuclei were stained using Hoechst 33342 (blue); (C) a merged image showing Hoechst 33342 staining and Texas Red staining.
(TIF)

Data S1 **Raw data for qPCR calculations, flow cytometry analysis, and ATP and NADH analysis.**
(XLSX)

Data S2 **Graphs in flow cytometry.**
(PPTX)

File S1 **Supporting Information.** Table S1, Primer sequences used for real-time PCR. Table S2, Detection of neuronal viability using the CCK-8 assay. Viability (%)  =  (ODs-OD_blank_)/(OD_0_-OD_blank_); ODs, the OD of each sample; OD_0_, the OD of NC cells incubated with 5 mM lysine (1.21±0.0503); OD_blank_, the OD of the blank control (0.23±0.0095). *P<0.05 vs. GA1 model cells (0.773±0.0208; survival rate, 55.4%). ^#^ *P*<0.05 vs. NC cells incubated in 5 mM lysine (survival rate, 100%).
(DOCX)
