# Supplementary figures and images for: Mechanistic Effects of Amino Acids and Glucose in a Novel Glutaric Aciduria Type 1 Cell Model
Source: PLoS One. 2014 Oct 15;9(10):e110181. doi: 10.1371/journal.pone.0110181 (PMC4198201; doi:10.1371/journal.pone.0110181)

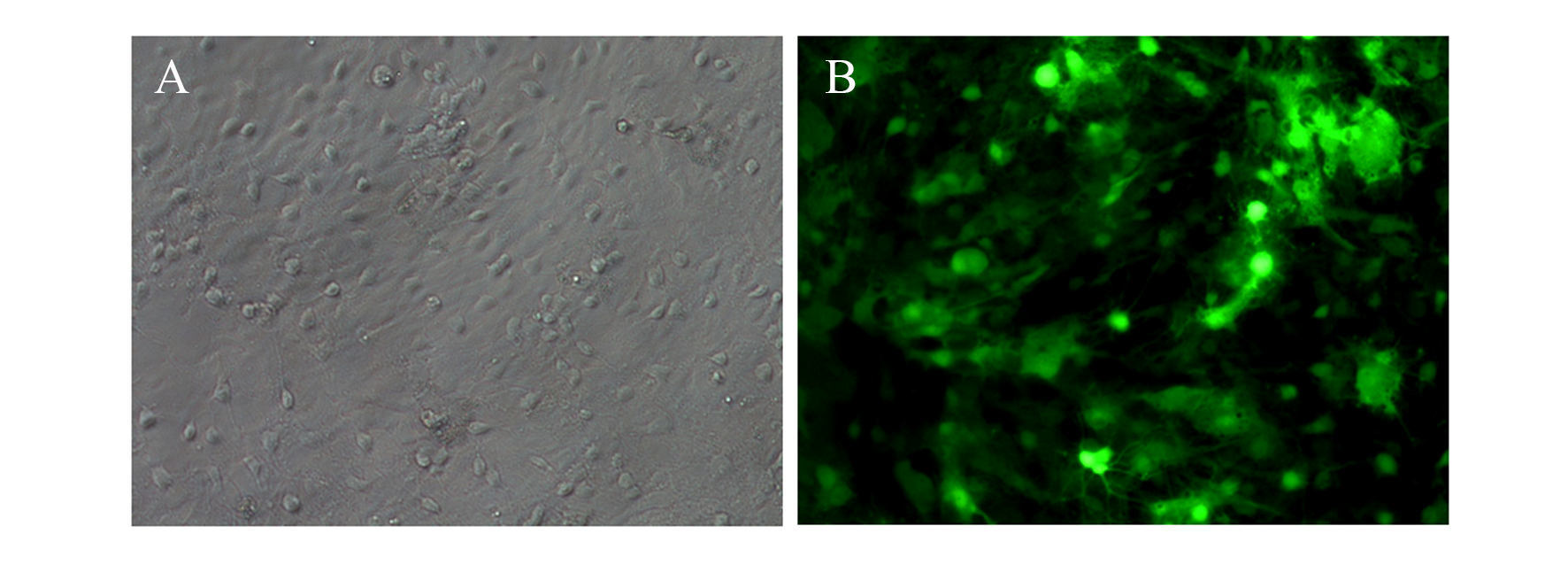

Supplement: Figure S1 — Striatal neurons infected with lentivirus. The striatal neurons were infected with the NC lentivirus at an MOI of 10; nearly all of the cells were infected and exhibited normal morphology. The observed fluorescence verified optimal infection conditions (magnification, 200×). (A) Bright-field microscopy of striatal neurons; (B) fluorescence microscopy of striatal neurons. (TIF) [file pone.0110181.s001.tif]

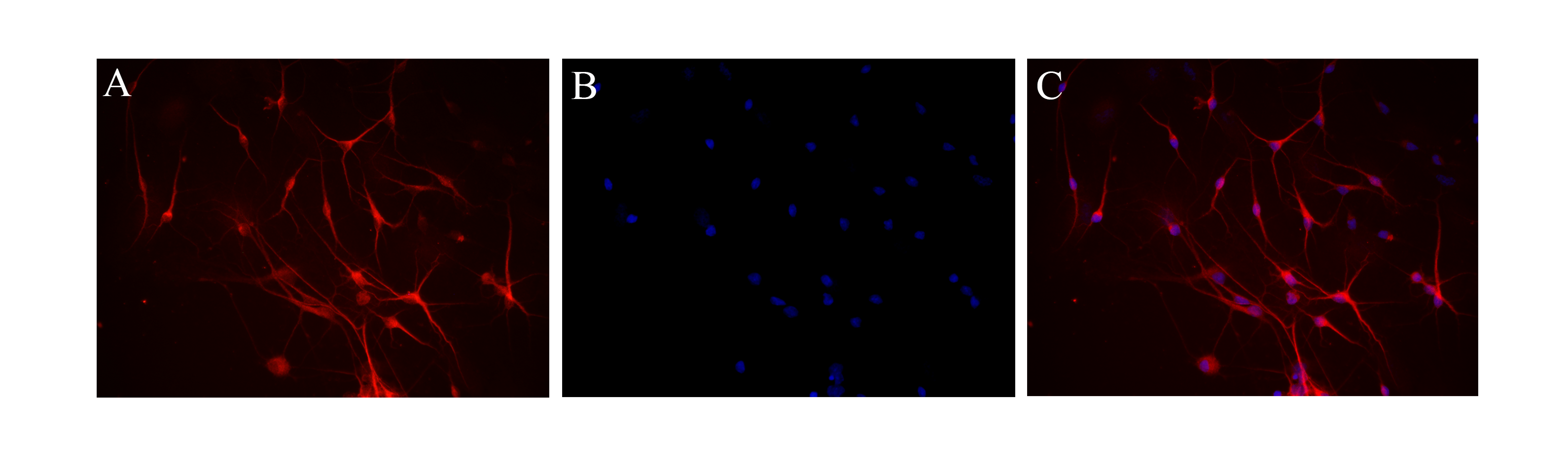

Supplement: Figure S2 — Assessment of neuronal purity. Immunofluorescence staining demonstrated the proportion of neurons in living cells to be greater than 90% (magnification, 200×). (A) Neuronal bodies and dendrites were labeled using Texas Red (red); (B) the cell nuclei were stained using Hoechst 33342 (blue); (C) a merged image showing Hoechst 33342 staining and Texas Red staining. (TIF) [file pone.0110181.s002.tif]
