# Supplementary material for: Mechanistic Effects of Amino Acids and Glucose in a Novel Glutaric Aciduria Type 1 Cell Model
Source: PLoS One. 2014 Oct 15;9(10):e110181. doi: 10.1371/journal.pone.0110181 (PMC4198201; doi:10.1371/journal.pone.0110181)
Supplement: Data S2 — Graphs in flow cytometry. (PPTX) [file pone.0110181.s004.pptx]

## Slide 1
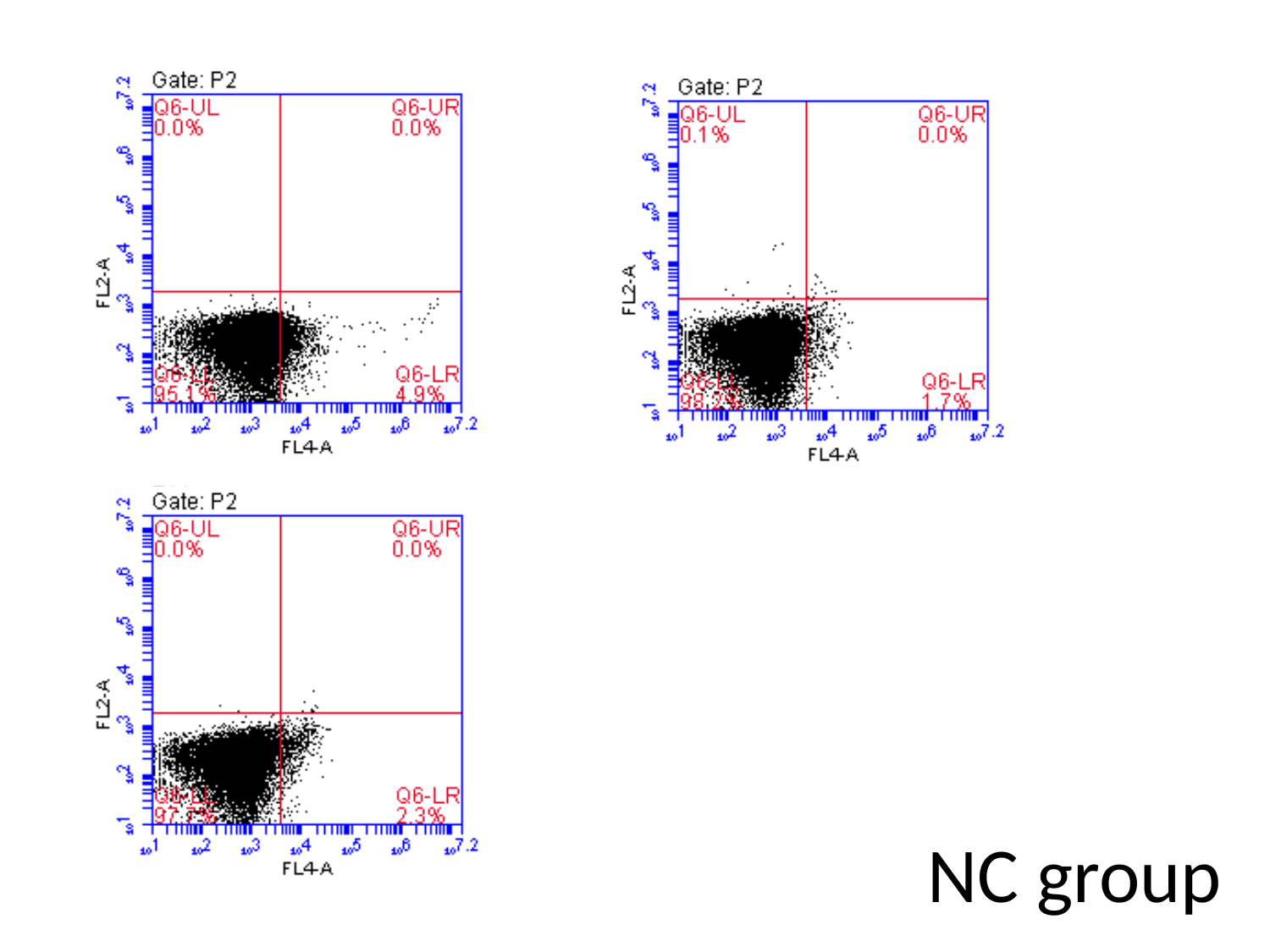

# NC group

## Slide 2
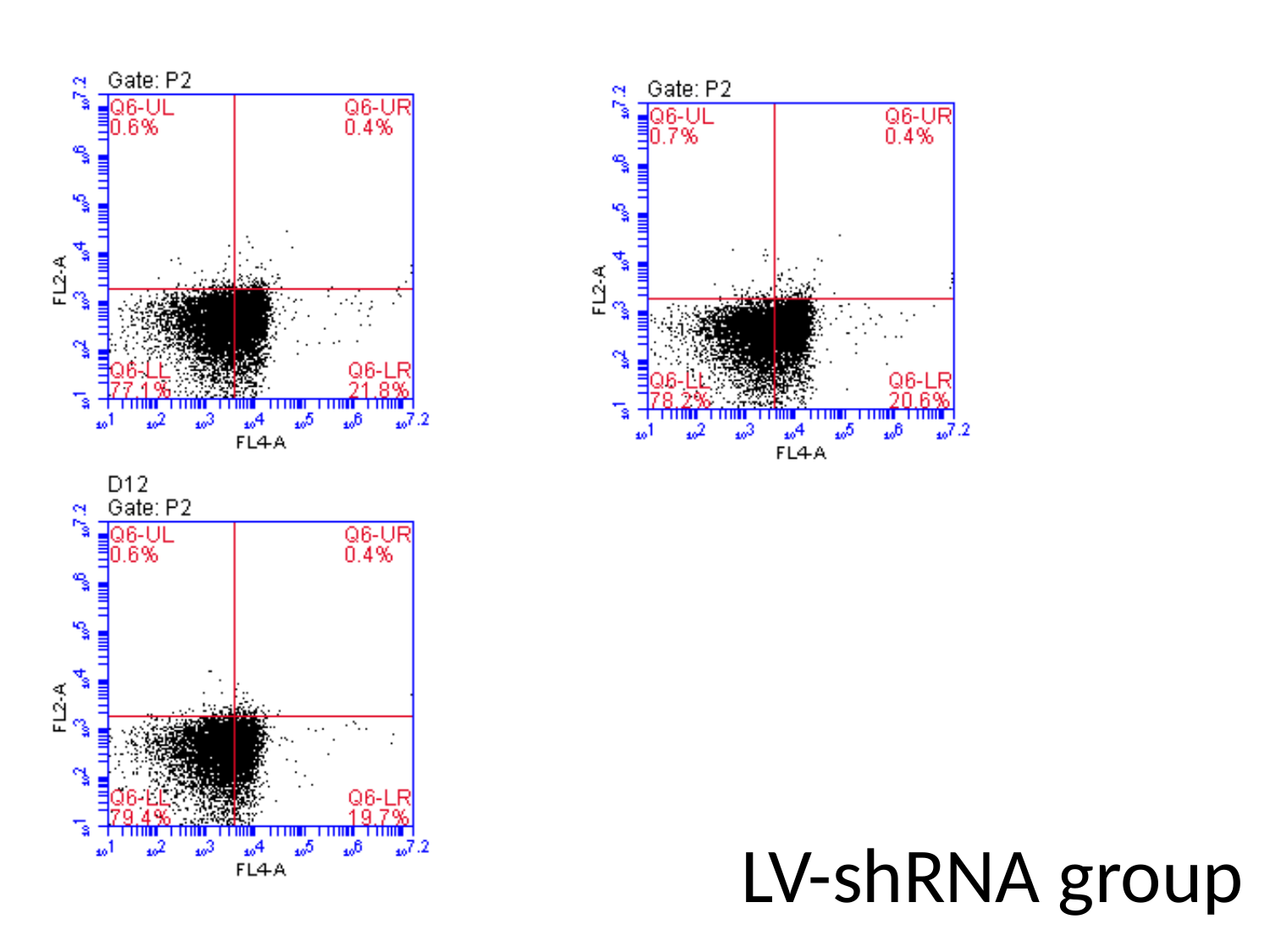

# LV-shRNA group

## Slide 3
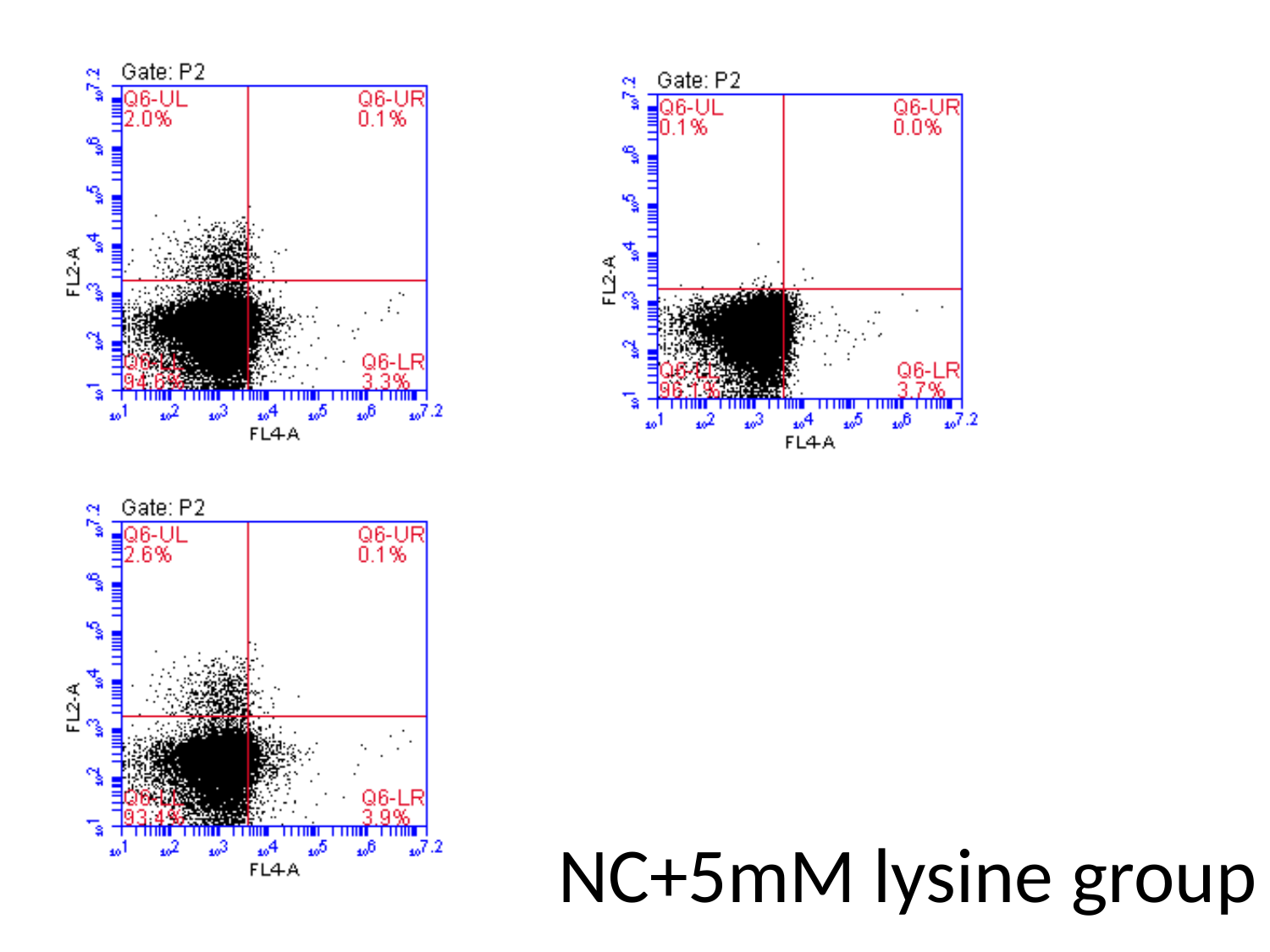

# NC+5mM lysine group

## Slide 4
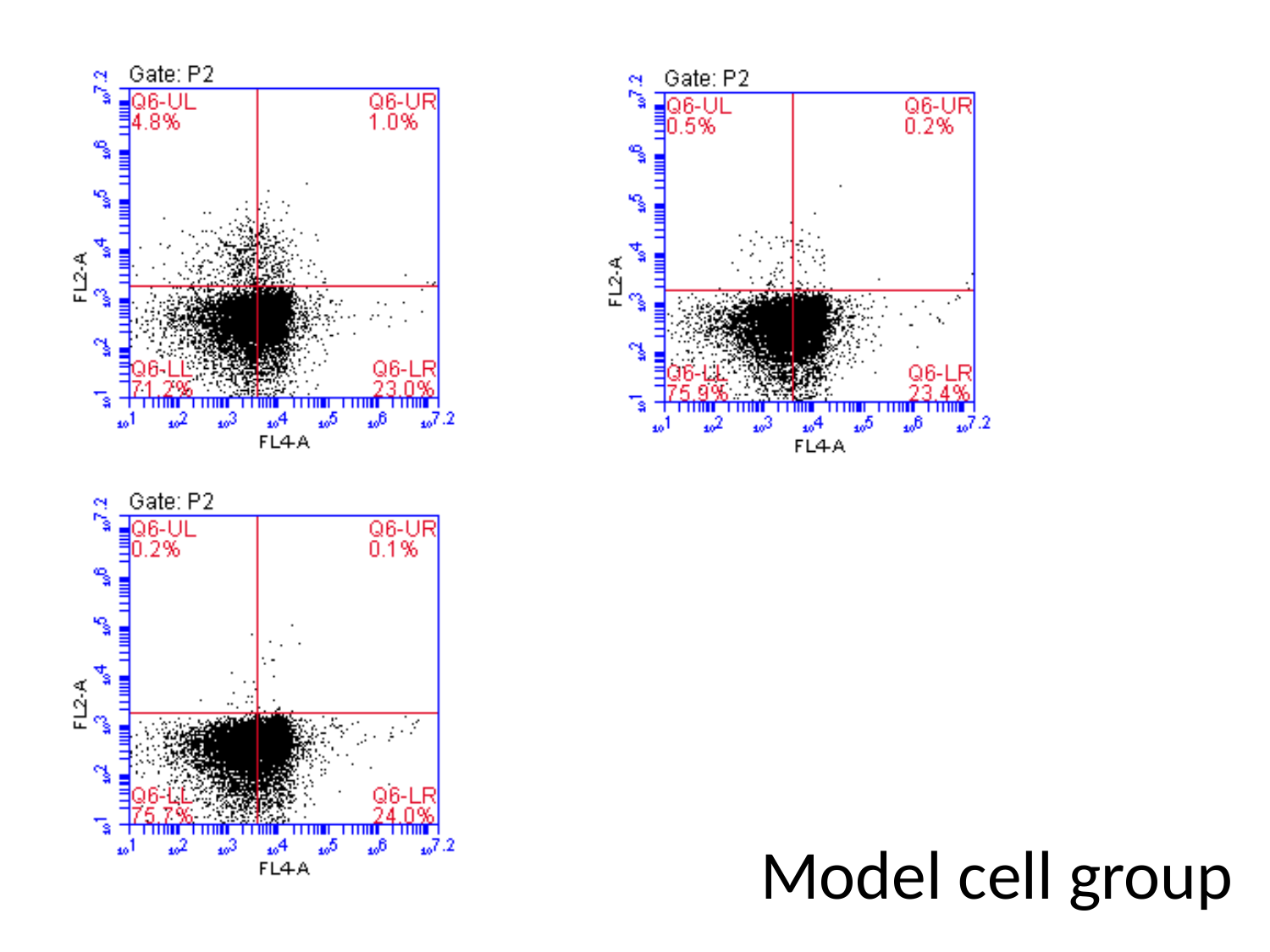

# Model cell group

## Slide 5
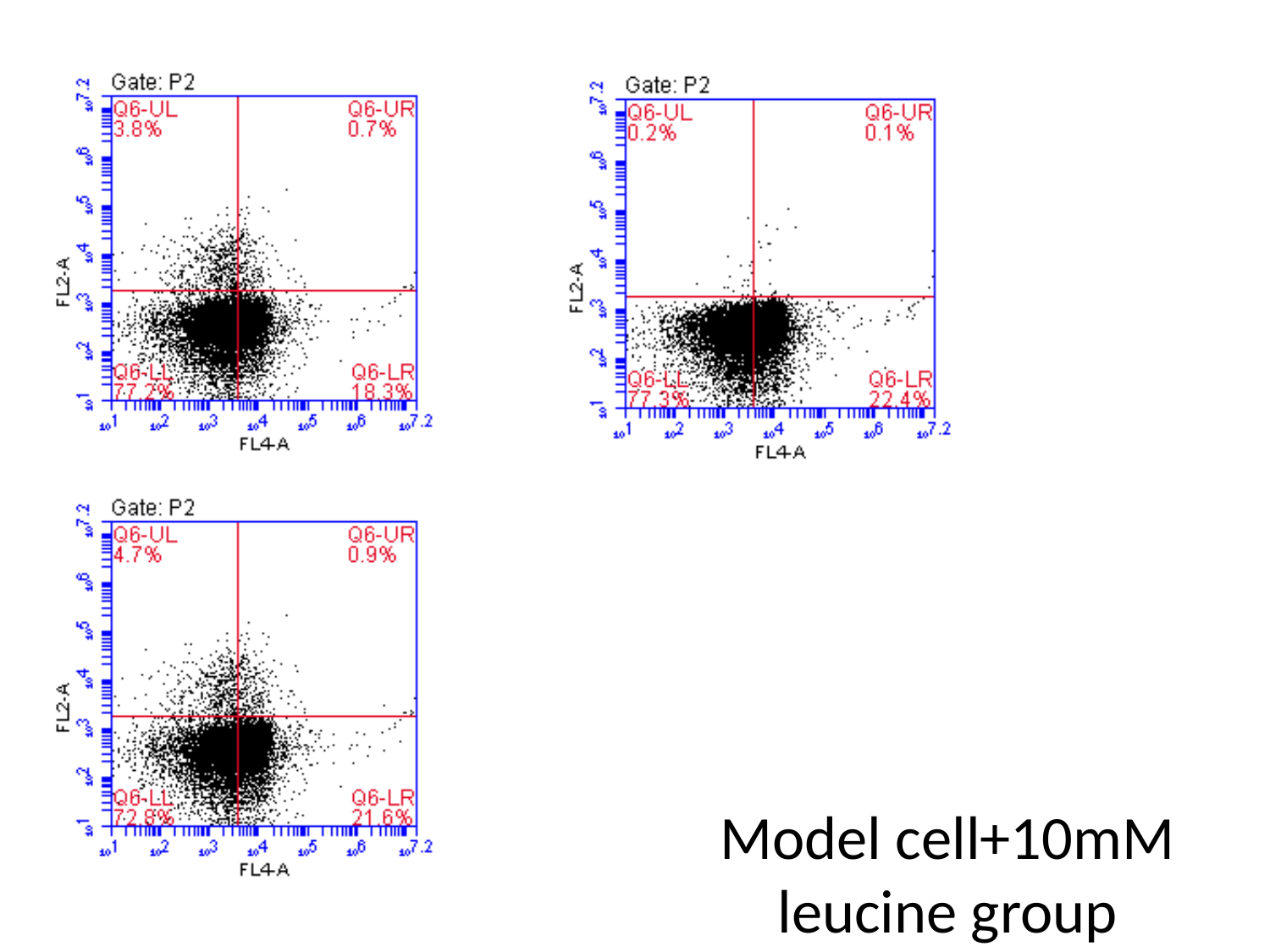

# Model cell+10mM leucine group

## Slide 6
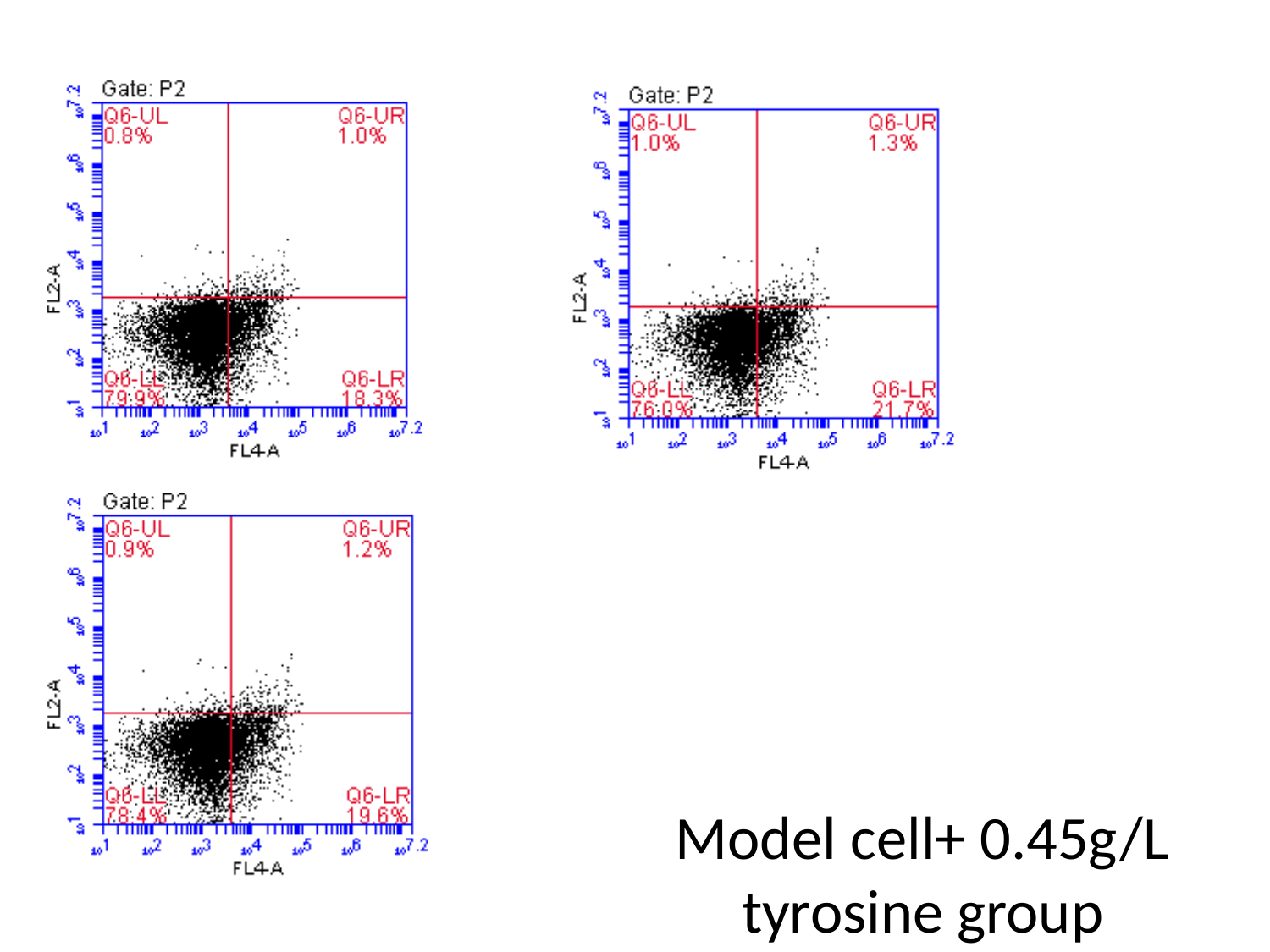

# Model cell+ 0.45g/L tyrosine group

## Slide 7
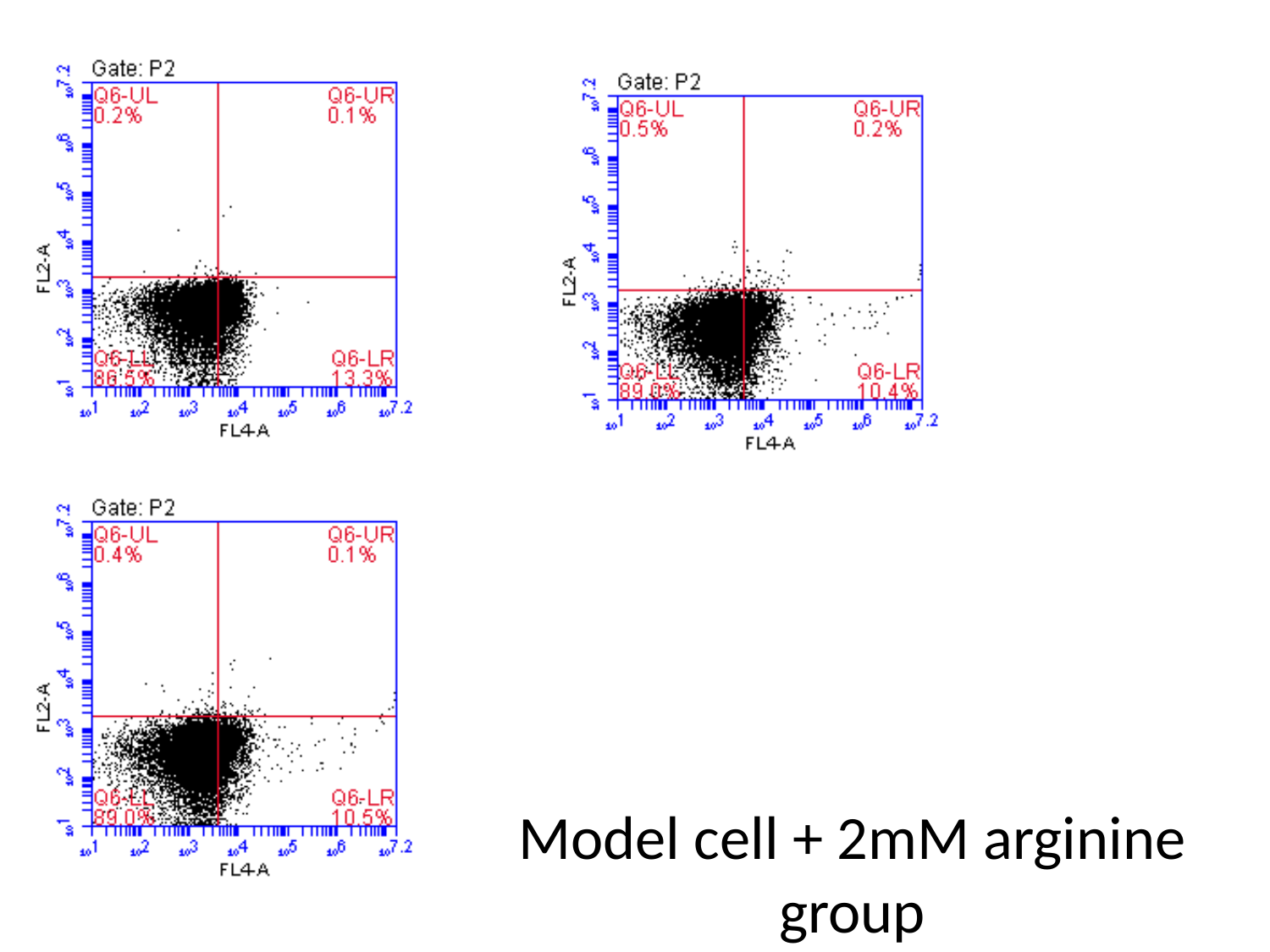

# Model cell + 2mM arginine group

## Slide 8
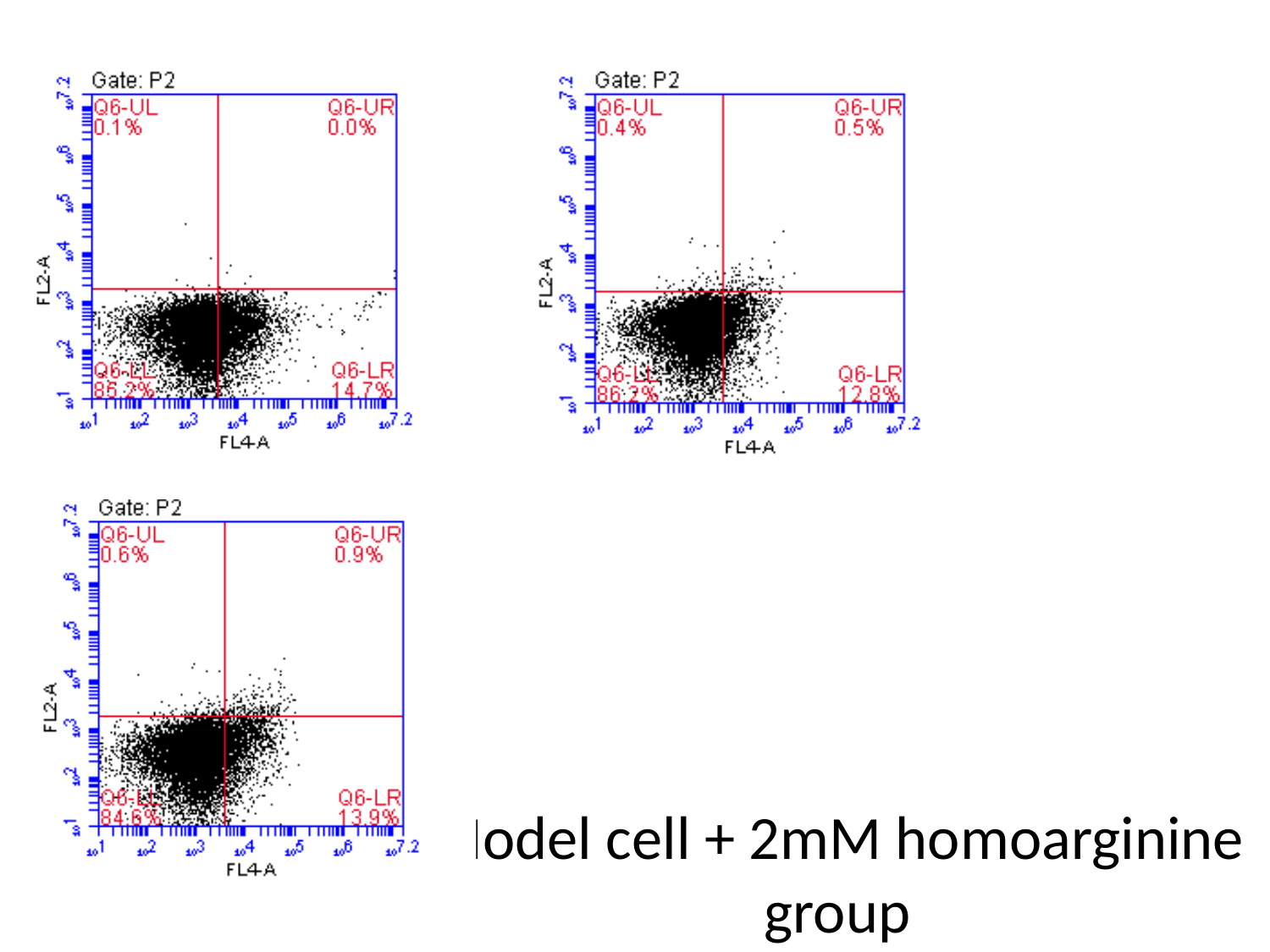

# Model cell + 2mM homoarginine group

## Slide 9
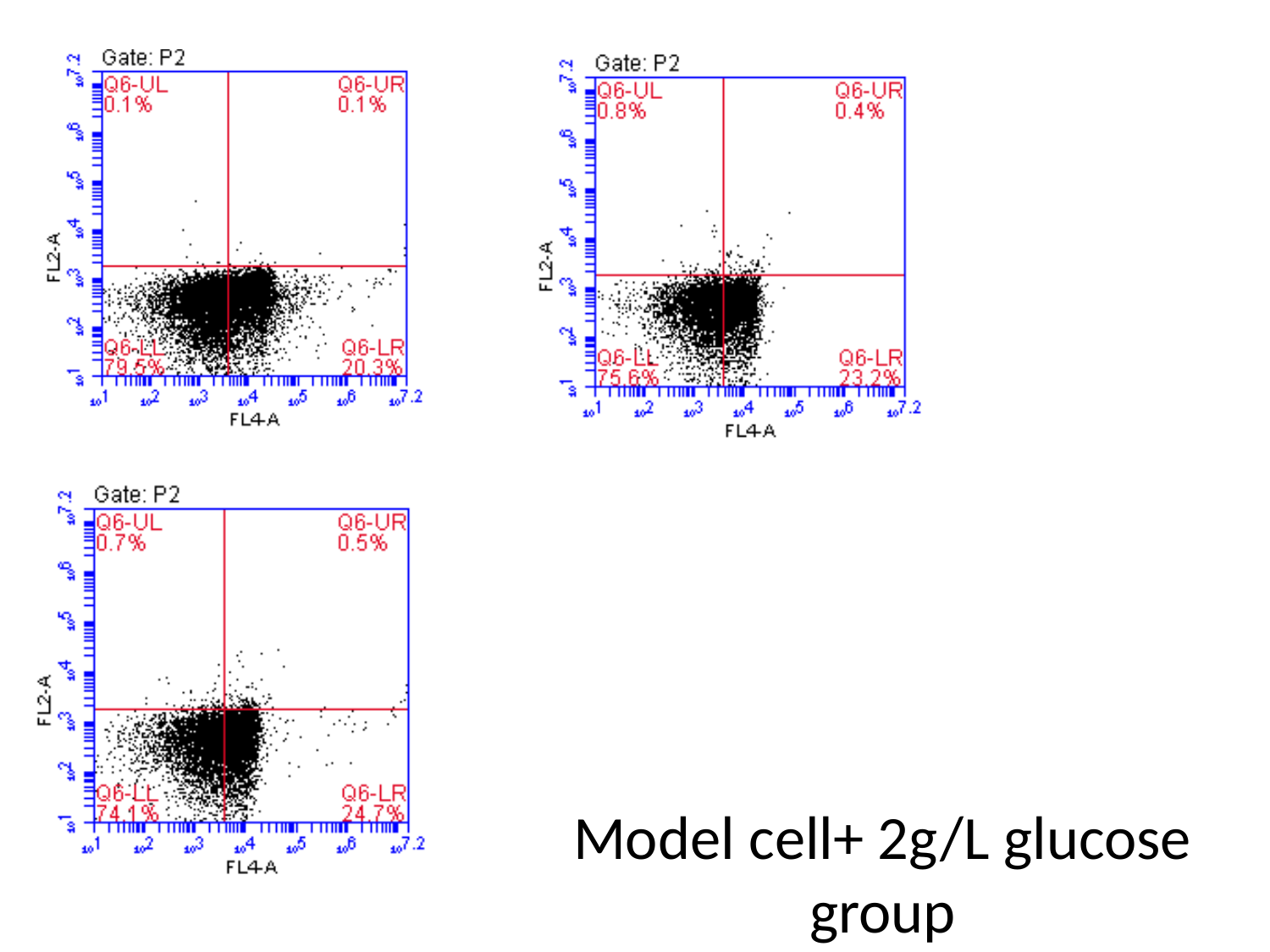

# Model cell+ 2g/L glucose group
