# Supplementary material for: Mechanistic Effects of Amino Acids and Glucose in a Novel Glutaric Aciduria Type 1 Cell Model
Source: PLoS One. 2014 Oct 15;9(10):e110181. doi: 10.1371/journal.pone.0110181 (PMC4198201; doi:10.1371/journal.pone.0110181)
Supplement: File S1 — Supporting Information. Table S1, Primer sequences used for real-time PCR. Table S2, Detection of neuronal viability using the CCK-8 assay. Viability (%) = (ODs-ODblank)/(OD0-ODblank); ODs, the OD of each sample; OD0, the OD of NC cells incubated with 5 mM lysine (1.21±0.0503); ODblank, the OD of the blank control (0.23±0.0095). *P<0.05 vs. GA1 model cells (0.773±0.0208; survival rate, 55.4%). # P<0.05 vs. NC cells incubated in 5 mM lysine (survival rate, 100%). (DOCX) [file pone.0110181.s005.docx]

**Supporting Information**

**Table S1. Primer sequences used for real-time PCR**

| **Primer** | **Sequence information** | **Length of amplified bands** | **GC percentage** |
| --- | --- | --- | --- |
| Caspase-3 Primer F | 5' GGCATCTCCTGTGATTGG 3' | 185 bp | 52% |
| Caspase-3 Primer R | 5' CTCAGCACTCTGGGAAAG 3' |  |  |
| Caspase-8 Primer F | 5'CTATCAAAGCAAGGACCACAAG 3' | 134 bp | 50% |
| Caspase-8 Primer R | 5'AGCCAGTGAAGTAAGATGTCAG 3' |  |  |
| Caspase-9 Primer F | 5' TGGTGGTGAGCAGAAAGAC 3' | 184 bp | 55% |
| Caspase-9 Primer R | 5'GAAGGTGGAGTAGGACACAAG 3' |  |  |
| Bcl-2 Primer F | 5' TGATAACCGGGAGATCGTG 3' | 133 bp | 56% |
| Bcl-2 Primer R | 5' AGGCTGGAAGGAGAAGATG 3' |  |  |
| Bax Primer F | 5' GGACGCATCCACCAAGAAG 3' | 134 bp | 55% |
| Bax Primer R | 5' CTGCCACACGGAAGAAGAC 3' |  |  |
| Fos Primer F | 5'GAACCCTTTGATGACTTCTTG 3' | 224 bp | 58% |
| Fos Primer R | 5'AAGGAAGACGTATAGGTAGTG3' |  |  |
| Jun Primer F | 5' CTGCCTTTGTAAGTTATTCC 3' | 230 bp | 32% |
| Jun Primer R | 5' TTCACCTAGCTCTCATTATC 3' |  |  |
| OGDC Primer F | 5'ACCCAGAGTCCCTTCAGTATG3' | 235 bp | 45% |
| OGDC Primer R | 5' GCGATTCCAATGCCAAACC 3' |  |  |
| FH Primer F | 5' ATGTCGCTGTTACTGTTG 3' | 242 bp | 44% |
| FH Primer R | 5' AATCTTCGCTGCTTTGTC 3' |  |  |
| PDC Primer F | 5' TCACGCCTATGCTACTGCTAAC 3' | 206 bp | 45% |
| PDC Primer R | 5'CCACAGCCACTGAAATGTCAAC3' |  |  |
| CS Primer F | 5' GTGCCAGAAACTGCTACC 3' | 111 bp | 54% |
| CS Primer R | 5' AGCCAAGAGACCTGTTCC 3' |  |  |
| GADPH Primer F | 5' GTCGGTGTGAACGGATTTG 3' | 181 bp | 51% |
| GADPH Primer R | 5' TCCCATTCTCAGCCTTGAC 3' |  |  |

**Table S2.Detection of neuronal viability using the CCK-8 assay**

|  | **GCDH-shRNA lentiviral vectors（+）and 5 mmol/L lysine（+）** | | | | | | | | |  |
| --- | --- | --- | --- | --- | --- | --- | --- | --- | --- | --- |
| **Arginine Con.** | | | **0.5 mM** | | **1 mM** | | **2 mM** | **5 mM** | **10 mM** |  |
|  | **OD** | | **0.782±0.0045**^#^ | | **0.854±0.0025***^#^ | | **0.887±0.0085***^#^ | **0.618±0.002***^#^ | **0.401±0.173***^#^ |  |
|  | **Viability** | | **56.3%** | | **63.6%** | | **67%** | **39.6%** | **17.4%** |  |
| **Homoarginine Con.** | | | **0.5 mM** | | **1 mM** | | **2 mM** | **5 mM** | **10 mM** | |
|  | **OD** | | **0.771±0.0125**^#^ | | **0.79±0.0005**^#^ | | **0.819±0.0175***^#^ | **0.672±0.0065***^#^ | **0.449±0.0135***^#^ | |
|  | **Viability** | | **55.1%** | | **57.1%** | | **60.1%** | **45.1%** | **22.3%** |  |
| **Glucose Con.** | | | **0.5 g/L** | | **1 g/L** | | **2 g/L** | **5 g/L** | **10 g/L** |  |
|  | **OD** | | **0.776±0.017**^#^ | | **0.78±0.0265**^#^ | | **0.784±0.011**^#^ | **0.782±0.037**^#^ | **0.589±0.1422**^#^ |  |
|  | **Viability** | | **55.7%** | | **56%** | | **56.5%** | **56.3%** | **36.6%** |  |
| **Leucine Con.** | | | | **1 mM** | | **5 mM** | **10 mM** | **20 mM** | **50 mM** |  |
|  | | **OD** | **0.763±0.0165**^#^ | | | **0.779±0.004**^#^ | **0.788±0.0155**^#^ | **0.775±0.0345**^#^ | **0.706±0.1575**^#^ |  |
|  | | **Viability** | | **54.3%** | | **56%** | **56.9%** | **55.6%** | **48.5%** |  |
| **Tyrosine Con.** | | | | **0.45 g/L** | |  |  |  |  |  |
|  | | **OD** | | **0.79±0.023**^#^ | |  |  |  |  |  |
|  | | **Viability** | | **57.1%** | |  |  |  |  |  |
